# The Use of Artificial Intelligence–Based Conversational Agents (Chatbots) for Weight Loss: Scoping Review and Practical Recommendations

**DOI:** 10.2196/32578

**Published:** 2022-04-13

**Authors:** Han Shi Jocelyn Chew

**Affiliations:** 1 Alice Lee Centre for Nursing Studies Yong Loo Lin School of Medicine National University of Singapore Singapore Singapore

**Keywords:** chatbot, conversational agent, artificial intelligence, weight loss, obesity, overweight, natural language processing, sentiment analysis, machine learning, behavior change, mobile phone

## Abstract

**Background:**

Overweight and obesity have now reached a state of a pandemic despite the clinical and commercial programs available. Artificial intelligence (AI) chatbots have a strong potential in optimizing such programs for weight loss.

**Objective:**

This study aimed to review AI chatbot use cases for weight loss and to identify the essential components for prolonging user engagement.

**Methods:**

A scoping review was conducted using the 5-stage framework by Arksey and O’Malley. Articles were searched across nine electronic databases (ACM Digital Library, CINAHL, Cochrane Central, Embase, IEEE Xplore, PsycINFO, PubMed, Scopus, and Web of Science) until July 9, 2021. Gray literature, reference lists, and Google Scholar were also searched.

**Results:**

A total of 23 studies with 2231 participants were included and evaluated in this review. Most studies (8/23, 35%) focused on using AI chatbots to promote both a healthy diet and exercise, 13% (3/23) of the studies used AI chatbots solely for lifestyle data collection and obesity risk assessment whereas only 4% (1/23) of the studies focused on promoting a combination of a healthy diet, exercise, and stress management. In total, 48% (11/23) of the studies used only text-based AI chatbots, 52% (12/23) operationalized AI chatbots through smartphones, and 39% (9/23) integrated data collected through fitness wearables or Internet of Things appliances. The core functions of AI chatbots were to provide personalized recommendations (20/23, 87%), motivational messages (18/23, 78%), gamification (6/23, 26%), and emotional support (6/23, 26%). Study participants who experienced speech- and augmented reality–based chatbot interactions in addition to text-based chatbot interactions reported higher user engagement because of the convenience of hands-free interactions. Enabling conversations through multiple platforms (eg, SMS text messaging, Slack, Telegram, Signal, WhatsApp, or Facebook Messenger) and devices (eg, laptops, Google Home, and Amazon Alexa) was reported to increase user engagement. The human semblance of chatbots through verbal and nonverbal cues improved user engagement through interactivity and empathy. Other techniques used in text-based chatbots included personally and culturally appropriate colloquial tones and content; emojis that emulate human emotional expressions; positively framed words; citations of credible information sources; personification; validation; and the provision of real-time, fast, and reliable recommendations. Prevailing issues included privacy; accountability; user burden; and interoperability with other databases, third-party applications, social media platforms, devices, and appliances.

**Conclusions:**

AI chatbots should be designed to be human-like, personalized, contextualized, immersive, and enjoyable to enhance user experience, engagement, behavior change, and weight loss. These require the integration of health metrics (eg, based on self-reports and wearable trackers), personality and preferences (eg, based on goal achievements), circumstantial behaviors (eg, trigger-based overconsumption), and emotional states (eg, chatbot conversations and wearable stress detectors) to deliver personalized and effective recommendations for weight loss.

## Introduction

### Background

The global prevalence of obesity has risen dramatically over the past 50 years and has now reached a state of a pandemic [1]. It was estimated that approximately 39% of the global adult population and more than 18% of the younger population were overweight in 2016 [2]. This creates a pressing public health concern because overweight and obesity increase one’s risk of disabilities, morbidities, and mortality from cardiometabolic diseases (eg, coronary artery disease and diabetes mellitus) [3], musculoskeletal disorders [4], cancers [5], and communicable diseases [6]. Having a high BMI have also been associated with a 32% increase in the likelihood of developing depression than having a normal weight, lowering one’s quality of life [7,8]. Although the prevalence of overweight and obesity is higher in adults, a meta-analysis reported that children and adolescents with obesity had a 5 times higher risk of transitioning to adulthood with obesity [9]. This highlights the importance of targeting both the adult and younger population in global weight management efforts.

Besides the minority cases where overweight and obesity are caused by pharmacological, metabolic, or genetic etiologies, people enrolled in weight loss programs are often prescribe diet (that reduces calorie intake) and exercise (that increases calorie expenditure) plans that create a state of prolonged calorie deficit. However, a major challenge of such interventions is the lack of adherence to restrictive lifestyle plans, often due to a lack of motivation and self-control (ie, cognitive inhibition: ability to control impulses) [10]. To overcome such challenges, health coaching has been shown to enhance the initiation and sustainability of weight loss efforts through nutrition and exercise education, goal setting, periodic progress monitoring, and positive encouragement [11]. However, such programs are labor intensive and resource inefficient [11,12]. Brief counseling techniques such as motivational interviewing have also been shown to improve one’s lifestyle behaviors but multiple empirical studies and systematic reviews have reported no significant superiority in interventional effectiveness when compared with other active comparators such as health coaching [13,14]. The findings were regardless of age and the mode of delivery [13-16], suggesting that current interventions are effective but impeded by their resource intensiveness (eg, time, manpower, and infrastructure) for coach training, program implementation, coordination, maintenance, and sustenance.

Recent technological advancements have enabled the use of computerized chatbots, also known as conversational agents (CAs), to mimic the role of human health coaches. Although terms such as chatbots, conversational artificial intelligence (AI), intelligence chatbots, and CAs are often used interchangeably, chatbots can be distinguished as those with and without AI [17]. In this paper, chatbots refer to computer software that is capable of having a conversation with someone and AI refers to the machinery mimicry of human intelligence to perform human tasks such as decision-making and problem solving, largely using machine learning [18]. Traditional rule-based chatbots without AI are only capable of identifying a limited number of client intents based on utterance interpretation of specific keywords [17]. This limits the degree of human conversation mimicry and hence the number of meaningful conversational turns to establish a motivational human-chatbot rapport. In contrast, AI chatbots are capable of machine learning to understand human intents and sentiments, thereby conversing with human-like demeanors to enhance human-chatbot interactions. This requires the use of natural language processing (NLP) for use cases such as natural language inference, sentiment analysis, and questioning and answering. In recent years, NLP has advanced from using traditional recurrent neural network models that analyze short texts for tasks such as summarization, translation, and abstraction to pretrained transformer models that analyze long texts as a whole to perform higher-level tasks of understanding and contextualization. Recent transformer models include Bidirectional Encoder Representations from Transformers by Google [19], Generative Pretrained Transformer (GPT-2 [20] and GPT-3 [21]) by Open AI, XLNet [22], and Turing Natural Language Generation by Microsoft [23]. The use of such technology in chatbots is more intuitive and able to express human emotions or cognitive responses such as empathy to enhance social presence, human-machine trust, emotional bond, user acceptability, and engagement [24]. A popular NLP platform used to develop and deploy such chatbots is Dialogflow, a user-friendly Google cloud-based platform capable of deploying text- and speech-based chatbots on various smartphone apps, websites, and Internet of Things (IoT) devices and appliances.

The use of AI in weight loss has been widely studied for its ability to efficiently and intuitively track diet, exercise, and energy balance. However, less is known about its ability to provide effective recommendations and behavioral nudges to enhance weight loss success [18]. Chatbots possess great potential as a communication vector for behavioral nudges through a sustained period of health coaching, thereby supplementing the role of a human health care professional in monitoring and counseling for weight loss. In addition, chatbots can provide 24/7 real-time monitoring, on-demand counseling, and personalized recommendation services conveniently through one’s preferred device and social communication platform (eg, WhatsApp, Telegram, and Facebook Messenger). Such functions have been shown to increase usability, user acceptability, engagement, and potential weight loss success because of their convenience and instantaneousness [25]. However, this is contingent upon the ability to forge a human-like rapport with users, which is one of the largest challenges in chatbot development. Moreover, little is known about the chatbots that have been developed to address health issues that require multiple long-term behavior changes such as for overweight and obesity [26].

### Objectives

This study aims to provide an overview of the potential use of AI chatbots for weight loss in people with overweight and obesity, and identify the essential components to prolong user engagement in AI chatbot–delivered weight loss programs. The term chatbot will hitherto refer to AI chatbots unless otherwise stated.

## Methods

This scoping review was performed according to the 5-stage framework by Arksey and O’Malley [27] and reported according to the PRISMA-ScR (Preferred Reporting Items for Systematic Reviews and Meta-Analyses extension for Scoping Reviews) checklist (Multimedia Appendix 1) [28].

### Stage 1: Identifying the Research Question

The research question for this study was developed based on the population, intervention, comparison, and outcomes framework, *What is known about the potential use of AI chatbots for weight loss in people with overweight and obesity and how can we prolong user engagement in AI chatbot–delivered weight loss programs?*

### Stage 2: Identifying Relevant Studies

The Cochrane Database of Systematic Reviews and PROSPERO databases were first searched to confirm that there was no previous systematic review on this topic. All studies published until July 9, 2021, were searched across nine databases: ACM Digital Library, CINAHL, Cochrane Central, Embase, IEEE Xplore, PsycINFO, PubMed, Scopus, and Web of Science. Keywords were permuted by iterative searching of PubMed and Medical Subject Headings terms using initial terms such as *chatbot* and *obesity*. The final search terms used were *overweight*, obes*, chatbot*, conversational agent*, virtual coach*, *artificial intelligence*, *machine learning*, and health coach*. The search strings connected using the Boolean operators are detailed in Multimedia Appendix 2. To ensure a comprehensive and extensive search on this topic, gray databases such as arXIV, Mednar, ProQuest Dissertation and Theses Global, and Science.gov were searched. Additional articles were also hand-searched from the reference lists of the included studies and the first 10 pages of Google Scholar.

### Stage 3: Study Selection

**Figure 1:**
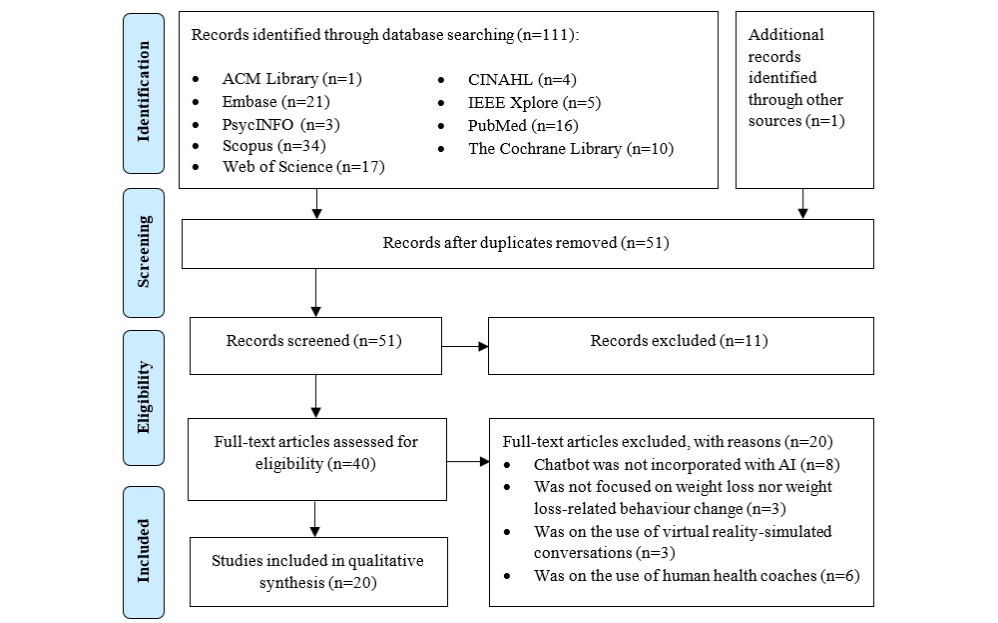
Flow diagram of the search strategy and search outcomes. AI: artificial intelligence.

The eligibility criteria for article inclusion were decided post hoc after an iterative screening of the resultant titles and abstracts and deeper familiarity with the topic. Articles that focused on the use of AI-based chatbots for weight loss were included. Given the lack of studies that focused on the use of AI chatbots for weight loss, population-based eligibility criteria such as age and weight status were not imposed to allow a discussion on the different needs of an AI chatbot tailored for populations with different demographics. Articles were excluded if they (1) used chatbots that did not incorporate AI (eg, chatbots and computerized coaches that were not conversational and without machine learning capabilities), (2) used human health coaches conversing with users through messaging platforms, (3) did not focus on weight loss or weight loss–related behavior change (eg, diet and exercise), and (4) were on virtual reality or simulation-based conversations and not real-life coaching. The search process and outcomes are shown in Figure 1.

### Stage 4: Charting the Data

Data extraction using Microsoft Excel was first pilot-tested for 3 studies and revised with additional headings before performing data extraction on all the included studies. The headings were author; year; country; type of publication; study design; participant characteristics; sample size; average age; proportion of male participants; baseline BMI; aims; name of the chatbot; delivery mode; use case; architecture; guiding framework; parameters collected; wearables or IoT; availability in multi-language; strategies used to improve user trust, rapport, or emotional connection with the chatbot; device for which the chatbot was deployed; machine learning algorithm or techniques; duration of weight loss program; outcome evaluation; engagement; acceptability; usability or usefulness; user suggestions; and key findings.

## Results

### Stage 5: Collating, Summarizing, and Reporting the Results

A total of 20 studies were included in this review, of which 1 study comprised 4 separate studies [29], resulting in 23 studies (representing 2231 participants) evaluated in this review. A summary and detailed description of the study characteristics are shown in Table 1 and Multimedia Appendix 3 [25,26,29-47] and Multimedia Appendix 4 [25,26,29-37,39-47]. The chatbot programs included Wakamola [30-32], WaznApp [33], WeightMentor [25], SWITCHes [34], MobileCoach [35], PathMate2 [36], and Lark Weight Loss Health Coach AI [37].

**Figure 2:**
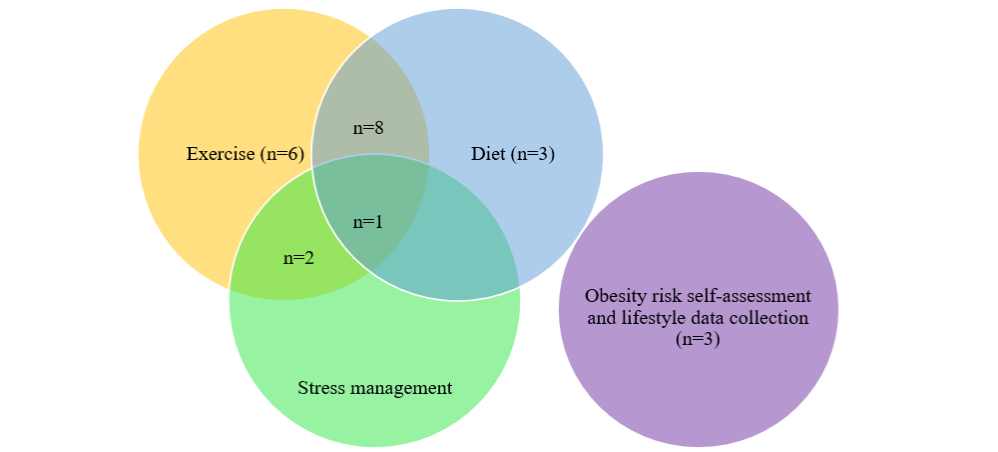
Summary of chatbot use cases for weight loss.

Most (8/23, 35%) of the studies focused on promoting a healthy diet and exercise, whereas only 4% (1/23) studies focused on a healthy diet, exercise, and stress management (Figure 2 and Multimedia Appendix 4). In all, 11 out of the 14 (79%) planned or trialed experimental studies [26,29-33,35-37,40,43-45] reported program durations that ranged from 1 hour to 12 months [30-32]. Only 1 study mentioned the intention of comparing algorithms to yield accurate behavioral predictions [43]. In total, 12 studies mentioned the use of a behavior change framework to guide AI chatbot development (Multimedia Appendix 4). A total of 3 studies used motivational interviewing [26,43,45]; 2 studies used cognitive behavioral therapy [37,45]; and others used mindfulness-based stress reduction [26], dialectic behavior therapy [41], efficiency model of support [39], and control theory by Carver and Scheier [34]. Moreover, 5 studies [29,33] referenced the use of taxonomy of behavior change techniques, whereas the remaining (9/23, 39%) studies did not specify the use of a structured behavior change framework (ie, briefly mentioned the incorporation of behavior change techniques such as goal setting, problem solving, and self-monitoring). Although the value of using a behavior framework to guide the development of weight loss chatbots remains unclear because of the limited number of publications derived from rigorous experimental studies, it could enhance the comprehensiveness of the developed programs and hence, the effectiveness of chatbots in addressing behavior change processes [48]. None of the studies explained the validation process such as using testing or training set splits or k-fold cross-validation.

**Table 1:** Summary of study characteristics (N=23).

Characteristics		Studies, n (%)
**Country**		
	Ireland [38]	1 (4)
	Italy [39]	1 (4)
	Lebanon [33]	1 (4)
	Spain [30-32]	3 (13)
	Switzerland [29,35,36,40]	7 (30)
	Taiwan [34]	1 (4)
	The Netherlands [41]	1 (4)
	United Kingdom [25,42]	2 (9)
	United States [26,37,43-46]	6 (26)
**Types of publication**		
	Conference [25,34,35,38-40,42-44]	9 (39)
	Internationally peer-reviewed journal articles [29-33,36,37,41,45,46]	14 (61)
**Study designs**		
	Developmental [25,34,35,38,39,43]	6 (26)
	Feasibility or pilot [26,30,31]	3 (13)
	N-of-1 longitudinal [29]	1 (4)
	Observational [32,37,45]	3 (13)
	Position or opinion paper [42,46]	2 (9)
	Protocol [33]	1 (4)
	Qualitative [29,41]	3 (13)
	Randomized controlled trials [36,40,44]	3 (13)
	Within-subject experiment [29]	1 (4)
**Participant characteristics**		
	Adults with a high BMI [26,37,41,42,44]	5 (22)
	Children and adolescents with a high BMI [35,36,45]	3 (13)
	General adults [25,29-33]	9 (39)
	General children and adolescents [39,40,43]	3 (13)
	NS^a^ [34,38,46]	3 (13)
**Sample sizes**		
	1-100 [25,26,29,31,35-37,40,41,43-45]	15 (65)
	100-800 [30,32]	2 (9)
	NS [33,34,36,38,39,42,46]	6 (26)
**Age (years)**		
	<18 [35-37,40,43]	5 (22)
	18-40 [26,29-32]	7 (30)
	41-65 [25,37,41,44]	4 (17)
	NS [29,33,34,38,39,42,46]	7 (30)
**Gender (male; %)**		
	0 [26,41]	2 (9)
	<50 [25,29-32,35,37,43-45]	11 (48)
	>50 [37,40,43]	3 (13)
	NS [29,33,34,38,39,42,46]	7 (30)
**Baseline BMI**		
	<25 kg/m^2^ [31,32]	2 (9)
	25-30 kg/m^2^ [25,26,30]	3 (13)
	>30 kg/m^2^ [41,44]	3 (13)
	>2 BMI-SDS^b^ (remaining studies on children and adolescents did not report BMI) [36,40]	2 (9)
	NS [29,33-35,38,39,42,43,45,46]	13 (57)
**Mode of delivery**		
	Speech [43,44]	2 (9)
	Text [25,30-33,35-37,39,40,42]	11 (48)
	Speech and text [34,45,46]	3 (13)
	Text and embodied conversational agent [26]	1 (4)
	Speech, text, and AR^c^-embodied conversational agent [29]	4 (17)
	NS [38,41]	2 (9)
**Multi-language**		
	Yes [30-32,34]	4 (17)
	NS [25,26,29,33,35-46]	19 (83)
**Incorporation of wearables or Internet of Things**		
	Yes [29,33,37,38,40,43]	9 (39)
	NS [25,26,30-32,34-36,39,41,42,44-46]	14 (61)
**Device used to operationalize chatbots**		
	Humanoid robot [43]	1 (4)
	Smartphone [25,29-31,33,34,36,40,42]	12 (52)
	Web browser [26]	1 (4)
	NS [32,35,37,38,41,44-46]	9 (39)
**Mention of machine learning techniques**		
	Yes [34,42,43,45]	4 (17)
	NS [25,26,29-33,35-41,44,46]	19 (83)
**Mention of behavior change framework**		
	Motivational interviewing [26,43,45]	3 (13)
	Cognitive behavioral therapy [37,45]	2 (9)
	Mindfulness-based stress reduction [26]	1 (4)
	Dialectic behavior therapy [41]	1 (4)
	Efficiency model of support [39]	1 (4)
	Control theory by Carver and Scheier [34]	1 (4)
	Taxonomy of behavior change techniques [29,33]	5 (22)

^a^NS: nonspecified.

^b^SDS: SD score.

^c^AR: augmented reality.

### Functions and Architecture

Core functions of the chatbot were to provide personalized weight loss recommendations (20/23, 87%) [25,26,29-34,37-39,41-46] and motivational messages (18/23, 78%) [25,26,29-33,37-39,41-43,45,46] (Multimedia Appendix 5 [25,26,29-37,39-47]). Only 26% (6/23) studies [30-32,36,40,43] mentioned the use of gamification to enhance user engagement, and 26% (6/23) studies [37,39,40,42,43,45] mentioned the use of sentiment analysis to provide emotional support through emotionally appropriate messages (Multimedia Appendix 5). These functions were generally achieved by (1) collecting various user-centric data through chatbot-based self-reports or device-detected metrics, (2) integrating collected parameters using machine learning techniques (including NLP to convert chatbot-collected information for prediction modeling) to predict and generate weight-related recommendations, (3) profiling users according to needs and preferences, and (4) providing personalized chatbot-delivered recommendations. The parameters collected included sociodemographic profiles (eg, age, race, ethnicity, education, work status, and income), food consumption, physical activity (eg, intensity, duration, frequency, and type), stress level, sleep (ie, duration), and clinical profiles (eg, presence of specific chronic diseases, medication use, smoking status, heart rate, and blood pressure; Multimedia Appendix 6 [25,26,29-37,39-47]). Majority of the parameters were collected through chatbots, except in 3 studies that estimated food consumption using smart refrigerator appliances [30] and nutritional information provided by retailers (scanning bar codes) [38,42]; 5 that estimated physical exercise type, intensity, and frequency wearable or smartphone sensors [33,37,38,42,43]; 2 that estimated stress levels using plasma cortisol and skin conductance response [36] and phone detection [37]; and 3 that measured heart rate and blood pressure [36,38,42]. Others that did not mention the use of wearables may have relied on information from in-built sensors of the phone. Only 4 studies elaborated on the algorithms and machine learning techniques used [25,39,42,45].

### Outcome Evaluations

Only 4 studies evaluated the effectiveness of a chatbot-delivered program on diet [26,37], physical activity [44], and weight loss [36] (Multimedia Appendix 3). Although 3 of these studies showed greater effectiveness in chatbot-delivered weight loss programs on the measured outcomes, 1 study reported that a higher proportion of adolescents in the control group lost weight as compared with those who interacted with the PathMate 2 chatbot (92% vs 61%). Those in the control group underwent 7 in-person counseling sessions with a health care professional, whereas those in the intervention group interacted with the PathMate 2 chatbot daily with 4 in-person counseling sessions (61%) [36]. Other studies (19/23, 83%) either used chatbots mainly to collect data on diet, physical activity, sitting time, and sleep [31,32] or were still in the developmental stage. Future studies should consider adopting an experimental design that evaluates the use of chatbots on objective weight-related outcomes such as weight loss, diet (eg, food choices, calorie intake, and consumption frequency), and physical activity (eg, energy expenditure, activity type, and activity frequency) using inferential statistics that suggest repeatability. Studies could also explore the mediation and/or moderation effects of these factors including user engagement and satisfaction on weight loss and weight loss maintenance as outcomes to examine the underlying mechanism by which AI chatbots influence weight loss.

### Engagement, Satisfaction, and Human-Chatbot Rapport

A total of 6 studies reported estimations of chatbot engagement that averaged at approximately 12 minutes a day [26,45], ranging from 4 minutes to 73 minutes per session [26,30,37,45]. The average daily app use was approximately 71% [36], with more than 4 conversational turns per day [40]. Measures of satisfaction in using the chatbots were heterogeneous, with estimates in terms of willingness to use [43], usability (eg, using the system usability scale) [30,35], adherence to recommendations [26,29,35,36], satisfaction (eg, 4 questions including a net promotor score) [37], and usefulness [45]. A summary of this section is provided in Multimedia Appendix 5.

An essential element of increasing chatbot engagement was the ability of the chatbot to form a human-chatbot rapport through interactivity and empathy (Multimedia Appendix 7 [25,26,29-37,39-47]). These required the system’s capacity to perform sentiment analysis for the interpretation and simulation of culturally appropriate human-like expression of verbal and nonverbal cues (for chatbots with embodiments, eg, speech intonations, facial expressions, and body language). Some techniques used were the deployment of humanoid robots [43] and embodied chatbots [26] that were capable of displaying visual social cues such as eye contact and hand gestures. Other techniques used in text-based chatbots were the delivery of colloquial, personally and culturally appropriate conversational tones and content [26,30]; emojis to emulate human emotional expressions [30-32]; positively framed words [32]; citations of credible information sources [26,33]; and validation (eg, acknowledgments and compliments) of not only behaviors but also thoughts and feelings [41]. Participants of the included studies were also found to have appreciated the personification of the chatbot (eg, funny, animated, empathetic, or playful) [25,29,31,32,37] and the provision of real-time, fast, and reliable recommendations [26,29,38]. In addition, studies (9/23, 39%) that enabled speech, instead of just text-based chatbot interactions (including those that use augmented reality [AR]) [29], improved engagement through a more convenient hands-free voice interaction with the chatbot [34,43-46]. This enabling of conversations through multiple platforms (eg, SMS text messaging, Slack, Telegram, Signal, WhatsApp, or Facebook Messenger) [45] and devices (eg, laptops, Google Home, and Amazon Alexa) [25] has also been reported to increase chatbot engagement because of greater convenience and access.

In contrast, users mentioned concerns regarding privacy and accountability [43,46], the inconvenience of having the chatbot on a limited number of third-party platforms (eg, only Telegram that one may not use) information [31], message or question overload that causes user fatigue [25,31], transparency about the app objectives and information sources [31], and appearing too robotic (eg, speaking too slowly in a robotic voice). Users also suggested that the chatbots should probe further to explore emotions and action plans instead of prescribing them [41]. Most strikingly, users suggested the incorporation of progress-based recommendations, rewards for goals achieved (ie, gamification), and integration with other tracking devices and appliances through IoT [25,26].

## Discussion

### Principal Findings

Overall, there is a strong potential in AI chatbot–delivered weight loss programs, but more studies are needed to assert sufficient evidence for its implementation in a population that is overweight and obese. The programs captured in this study were heterogeneous in their weight loss use cases, functions, architecture, mode of delivery, and interoperability with other devices and databases. This highlights the need for further research on the impact of various chatbot features such as gamification, personification, and the ability to express empathy and to design and develop an efficient system for weight loss. Most (6/23, 26%) of the studies were still in the development phase (including feasibility testing and qualitative studies on needs and perceptions), with only 3 randomized controlled trials that only reported favorable outcomes of the chatbot on interim user engagement [40], increasing physical activity [44], and weight loss [36]. Only 35% (8/23) of the studies focused on participants with overweight and obesity, 4% (1/23) of the studies were conducted in an Asian context, and most of the studies had a small sample size (15/23, 65%). These gaps raise questions on the receptibility, applicability, and effectiveness of AI chatbots in weight-related behavior change and weight loss in populations with different demographics such as age, weight status, and culture. Among the included studies, 1 study (1/23, 4%) reported that a higher proportion of adolescents in the control group who underwent 7 in-person counseling sessions lost weight as compared with the intervention group who interacted daily with the PathMate 2 chatbot [36]. This finding was contrary to the other 3 studies that reported better diet and exercise improvements in adults who interacted with a chatbot [26,37,44]. Assuming that the improvements in diet and exercise were extrapolated to an eventual weight loss that was not evaluated in the 3 studies, this discrepancy could be associated with adolescents having a lower self-regulation capacity than adults, indicating that chatbot designs must be age appropriate [49]. Having a lower self-regulation capacity may suggest that one requires more frequent and in-person support for impulse control (eg, succumbing to dietary temptations) rather than communicating with a chatbot that is easy to ignore when one is unmotivated. Therefore, chatbot designs for children and adolescents may require more attention-grabbing features such as having an animated embodied CA, more interactivity (ie, engaging as many of the 5 senses as possible) possibly through AR, and gamification to sustain program engagement [50].

Most studies highlighted the use of chatbots to provide personalized nutrition and exercise recommendations and motivational messages, but few studies mentioned the use of gamification and sentiment analysis. Weight loss mobile health apps such as My Fitness Pal and Lifesum are often embellished with gamification features to improve motivation, user engagement, and program effectiveness toward health behavior changes. A study on the 50 most downloaded health apps on the App Store reported that 64% of such apps included some form of goal setting, social presence, challenge, monetary, and social (eg, accomplishing challenges and gaining points to reach higher competition grading tiers) incentives [51]. However, studies have shown that such gamification features do not result in significantly different amounts of weight loss at 3, 6, 9, or 12 months between adults who do and do not undergo such programs [52,53]. Similarly, a meta-analysis reported that gamification did not result in significant weight loss differences between children and adolescents who did and did not undergo gamification for weight loss, although those in the former group were found to have improved nutritional knowledge scores [54]. This suggests that although gamification may improve weight loss knowledge, user engagement, and intention toward health behavior change, it is insufficient to impact any actual weight loss. Therefore, future studies should focus on identifying more practical and core reasons for weight loss failure, such as the inability to control food temptations, and capitalize on AI chatbot technology to provide real-time nudges.

Major challenges in app-delivered weight loss programs, especially for people with overweight and obesity, lie in users’ motivation and discipline toward a diet and exercise regime [55]. Personalization recommendations are well known to enhance goal attainment; hence, mobile health apps strive to provide recommendations based on one’s demographic profile, anthropometric status, and monitored calorie intake and output [56]. However, recent studies have shown that this level of personalization is insufficient to sustain weight loss behavior change and that some form of emotional support is required [56]. This is because of the common weight loss–related experiences of stigmatization, self-loathing, and social shaming, which evoke negative emotions such as guilt, shame, self-reproach, regret, depression, anxiety, low self-esteem, and stress [47,57,58]. Such negative emotions could also create a vicious cycle of increasing weight gain, as one copes with such negative emotions by seeking comfort in food. Consistently, poor emotional regulation has been associated with weight regain and weight loss failure, regardless of age, despite the use of behavioral regulation strategies [59,60]. However, current clinical and commercial weight loss programs often neglect this aspect of weight loss, possibly because of the more complex and time-consuming nature. Therefore, interventions that provide emotional support such as health coaching could improve weight loss by forging a supportive coach-client relationship that provides on-demand emotional and knowledge support through accountability, compassion, and empathy [61,62]. However, health coaches are resource intensive and burden the health care system, and the use of AI has been shown to reduce health care costs by increasing health care service delivery efficiencies [63]. Therefore, AI chatbots could supplement the function of health coaches at a lower annualized health care expenditure (eg, through more accurate weight predictions and recommendations, reduced man-hours and infrastructure needed, and reduced admissions). However, this requires chatbots to have enhanced abilities to track emotions through sentiment analysis and emotional modeling to provide empathetic, context-specific messages to motivate health behavior changes, especially in vulnerable situations (eg, in the circumstance of food temptation) [64]. Only 6 of the included studies mentioned the use of sentiment analysis to provide more human-like conversations that consider emotions, and further research is needed to evaluate its effectiveness in improving user engagement and weight loss. The included studies also highlighted some innovative features used to enhance the likeliness of human-like verbal and nonverbal cues such as providing culturally appropriate conversation content; incorporating interactivity and relatability through animations and personified embodied chatbots; and conveying emotions through emojis and body gestures. More research is also needed to evaluate the effects of AI chatbot delivery mode, namely, text-based, speech-based (eg, Alexa), visual (animated 2D characters), and AR-based (animated 3D characters) CAs on user engagement, behavior change, and weight loss.

### Practical Recommendations

Overall, chatbots can be programmed to (1) fetch information (eg, weight status, food consumption, and exercise) through conversations with users (eg, asking about food consumed and exercises performed), multiple databases (eg, electronic medical records), devices (eg, activity trackers and smartphones), and smart appliances (eg, smart refrigerators and motion sensors); (2) integrate such information to optimize predictive models of weight loss; (3) synthesize personalized weight loss plans; and (4) provide real-time adaptive recommendations (eg, decision-making and self-regulation skills training), progress feedback (eg, how much more exercise to do to reach a certain weight loss goal by a stipulated time), and emotional support (eg, motivation, empowerment, and validation) through conversations with users.

### Limitations

Certain relevant evidence could have been precluded from this study, undermining the comprehensiveness of this review, although many databases including gray literature were searched. This includes programs that were commercialized and marketed without a research study and studies published in other languages. Studies included in this review were also largely heterogeneous in study design, participant characteristics, and outcomes measured, impeding the comparisons between AI chatbot elements, weight-related outcome measures, and architectures to inform future chatbot designs and developments. However, this also highlights the infancy and potential of such technology in reducing the health care burden of overweight and obesity, a long-standing public health problem.

### Conclusions

This study highlighted the potential of AI chatbots in providing just-in-time personalized weight loss–related behavior change recommendations, motivational messages, and emotional support. These require the integration of a comprehensive set of information beyond the conventional health metrics from self-reports, app trackers, and fitness wearables. This includes personality and preferences (eg, based on goal achievements), circumstantial behaviors (eg, trigger-based overconsumption), and emotional states (eg, chatbot conversations and wearable stress detectors). AI chatbots should be designed to be human-like, personalized, contextualized, immersive, and enjoyable to enhance user experience, engagement, behavior change, and weight loss. Future AI chatbot developments should also consider issues of privacy; accountability; user burden during chatbot engagement; and interoperability with other databases (eg, electronic medical records), third-party apps (eg, health tracking apps), social media platforms (eg, data mining from Twitter, Facebook, and Instagram posts), devices (eg, laptops, desktops, and phones), and appliances (eg, refrigerators and gaming consoles). Future AI chatbots should also be designed as a one-stop diet, exercise, and emotional support app to derive at a market-ready and effective chatbot-delivered weight loss program.

## Appendix

Multimedia Appendix 1 PRISMA-ScR (Preferred Reporting Items for Systematic Reviews and Meta-Analyses extension for Scoping Reviews) checklist.

Multimedia Appendix 2 Details on search terms used for each database.

Multimedia Appendix 3 Detailed characteristics of each study (N=23).

Multimedia Appendix 4 Use cases, names, delivery modes, deployment devices, wearables or Internet of Things and behavioral framework of the conversational agents used in the included studies (N=23).

Multimedia Appendix 5 Architecture or descriptions and core functions of conversational agents used in the included studies (N=23).

Multimedia Appendix 6 Parameters collected from users to develop conversational agent-based weight loss interventions (N=23).

Multimedia Appendix 7 Engagement, satisfaction and human-conversational agent emotional connection described in the included studies (N=23).

## Abbreviations

AI: artificial intelligence
AR: augmented reality
CA: conversational agent
IoT: Internet of Things
NLP: natural language processing
PRISMA-ScR: Preferred Reporting Items for Systematic Reviews and Meta-Analyses extension for Scoping Reviews
