# Supplementary material for: The Use of Artificial Intelligence–Based Conversational Agents (Chatbots) for Weight Loss: Scoping Review and Practical Recommendations
Source: JMIR Med Inform. 2022 Apr 13;10(4):e32578. doi: 10.2196/32578 (PMC9047740; doi:10.2196/32578)
Supplement: Multimedia Appendix 2 [file medinform_v10i4e32578_app2.docx]

**Appendix 2:** Details on search terms used for each database.

| Topic |  | Search terms | Number of studies |
| --- | --- | --- | --- |
| ACM Digital Library | 1 | [[All: "artificial intelligence"] OR [All: "machine learning"]] AND [Abstract: "health coach*"] | 7 |
|  | 2 | [Abstract: chatbot*] OR [Abstract: "conversational agent*"] OR [Abstract: "virtual coach*"] | 598 |
|  | 3 | [Abstract: overweight] OR [Abstract: obes*] | 325 |
|  | 4 | [[[[Abstract: "artificial intelligence"] OR [Abstract: "machine learning"]] AND [Abstract: "health coach*"]] OR [Abstract: chatbot*] OR [Abstract: "conversational agent*"] OR [Abstract: "virtual coach*"]] AND [[Abstract: overweight] OR [Abstract: obes*]] | **1** |
| CINAHL | 1 | AB ( "artificial intelligence" OR "machine learning" ) AND AB "health coach*" | 3 |
|  | 2 | ( AB ( "artificial intelligence" OR "machine learning" ) AND AB "health coach*" ) OR AB ( chatbot* OR "conversational agent*" OR "virtual coach*") | 267 |
|  | 3 | ( ( AB ( "artificial intelligence" OR "machine learning" ) AND AB "health coach*" ) OR AB ( chatbot* OR "conversational agent*" OR "virtual coach*") ) AND AB ( overweight OR obes* ) | **4** |
| The Cochrane Library  IEEE xplore | 1 | ("artificial intelligence" OR "machine learning") AND ("health coach*"):ti,ab,kw | 4 |
|  | 2 | (chatbot* OR "conversational agent*" OR "virtual coach*"):ti,ab,kw | 134 |
|  | 3 | #1 OR #2 | 138 |
|  | 4 | (overweight OR obes*):ti,ab,kw | 48,462 |
|  | 5 | #3 AND #4 | **10** |
| Embase | 1 | ('artificial intelligence' OR 'machine learning') AND 'health coach*':ab,ti | 19 |
|  | 2 | chatbot* OR 'conversational agent*' OR 'virtual coach*' | 512 |
|  | 3 | #1 OR #2 | 525 |
|  | 4 | overweight:ab,ti OR obes*:ab,ti | 507557 |
|  | 5 | #3 AND #4 | **21** |
| IEEE Xplore | 1 | ("Full Text & Metadata":"artificial intelligence" OR "Full Text & Metadata":"machine learning") AND ("Abstract":"health coach*") | 7 |
|  | 2 | ("Abstract":chatbot* OR "Abstract":"conversational agent*" OR "Abstract":"virtual coach*") | 901 |
|  | 3 | ("Abstract":overweight OR "Abstract":obes*) | 1145 |
|  | 4 | ((("Full Text & Metadata":"artificial intelligence" OR "Full Text & Metadata":"machine learning") AND ("Abstract":"health coach*") OR ("Abstract":chatbot* OR "Abstract":"conversational agent*" OR "Abstract":"virtual coach*")) AND("Abstract":overweight OR "Abstract":obes*) ) | **5** |
| PubMed | 1 | (overweight[Title/Abstract] OR obes*[Title/Abstract]) AND (("conversational agent*"[Title/Abstract] OR chatbot[Title/Abstract] OR "virtual coach*"[Title/Abstract]) OR (("artificial intelligence" OR "machine learning") AND ("health coach*"[Title/Abstract]))) | **16** |
| PsycINFO | 1 | ("artificial intelligence" or "machine learning").af. and "health coach*".ab. | 3 |
|  | 2 | (chatbot* or "conversational agent*" or "virtual coach*").ab. | 358 |
|  | 3 | (("artificial intelligence" or "machine learning").af. and "health coach*".ab.) or (chatbot* or "conversational agent*" or "virtual coach*").ab. | 361 |
|  | 4 | ((("artificial intelligence" or "machine learning").af. and "health coach*".ab.) or (chatbot* or "conversational agent*" or "virtual coach*").ab.) and (overweight or obes*).ab. | **3** |
| Scopus | 1 | ( ALL ( "artificial intelligence" OR "machine learning" ) AND TITLE-ABS-KEY ( "health coach*" ) ) | 70 |
|  | 2 | TITLE-ABS-KEY ( chatbot* OR "conversational agent*" OR "virtual coach*" ) | 5865 |
|  | 3 | ( ( ALL ( "artificial intelligence" OR "machine learning" ) AND TITLE-ABS-KEY ( "health coach*" ) ) OR TITLE-ABS-KEY ( chatbot* OR "conversational agent*" OR "virtual coach*" ) ) AND TITLE-ABS-KEY ( overweight OR obes* ) | **34** |
| Web of Science | 1 | "artificial intelligence" OR "machine learning" (All Fields) and "health coach*" (Abstract) | 16 |
|  | 2 | ((AB=(chatbot* OR "conversational agent*" OR "virtual coach*" )) OR #1) | 2513 |
|  | 3 | (((AB=(chatbot* OR "conversational agent*" OR "virtual coach*" )) OR #1)AND AB=(overweight OR obes*)) | **17** |
|  |  | Total | 111 |
