# Supplementary material for: The Use of Artificial Intelligence–Based Conversational Agents (Chatbots) for Weight Loss: Scoping Review and Practical Recommendations
Source: JMIR Med Inform. 2022 Apr 13;10(4):e32578. doi: 10.2196/32578 (PMC9047740; doi:10.2196/32578)
Supplement: Multimedia Appendix 3 [file medinform_v10i4e32578_app3.docx]

**Appendix 3.** Detailed characteristics of each study (n=23)

| **Author, year** | **Aims** | **N** | **Mean age** | **% male** | **Baseline BMI** | **Key findings** |
| --- | --- | --- | --- | --- | --- | --- |
| Addo, et al., 2013 [43] | To present the design and prototype implementation of a collective intelligence approach that facilitates personalization of a humanoid robot Health Coach for childhood obesity intervention. | 15 | Range: 3-19 | 53 | NS | Human-Robot Interaction (HRI) could improve health behavior change. |
| Asensio-Cuesta, et al., 2021a [30] | To assess the feasibility of using a user-centered chatbot for collecting linked data to study overweight and obesity causes in a target population. | 980 | 30.1 | 37 | 25.8 | Wakamola´s food consumption results could support the detection of nutritional problems in a population, directing the population´s nutritional recommendations and focusing effective intervention programs at the population level. |
| Asensio-Cuesta, et al., 2021b [31] | To report the user-centered design and feasibility of a chatbot to collect linked data to support the study of individual and social overweight and obesity causes in populations. | 74 | 20.7 | 27 | 21.4 | Wakamola is a feasible tool to collect data from a population about socio-demographics, diet patterns, physical activity, BMI, specific diseases and social networks. The most consumed foods were olive oil, milk and derivatives, cereals, vegetables, and fruits. People walked 10 minutes on 5.8 days per week, slept 7.02 hours per day, and were sitting 30.57 hours per week. |
| Asensio-Cuesta, et al., 2021c [32] | To study the effect of confinement on weight and lifestyle using the Wakamola chatbot. | 739 | 28.6 | 40 | 23.2 | Wakamola helped to contact participants and confirm changes in their lifestyle during confinement. Nutrition score improved for men (medians 81.77–82.29, p < 0.05), with no difference for women (medians 82.29 in both cases). Both genders reduced their physical activity score (men 100–40.14, p < 0.01, women 80.42–36.12, p < 0.01). Women sat less hours/week, men’s medians 28.81–28.27, women’s medians 35.97–23.33, p = 0.03. Both genders slept longer (hours/day), men 7–7.5, women 7–8 (p < 0.01) (medians). |
| Bardus, et al., 2018 [33] | To examine the feasibility of a self-directed weight loss intervention using a virtual coaching app with JITAI features (Lark) and a self-help calorie-counting app (MyFitnessPal). | NS | NS | NS | NS | NS (This was a study protocol) |
| Dol, et al., 2021[41] | To explore the opportunities for helping adults with emotional overeating using a virtual coach, aiming to identify preferences for tailored coaching strategies applicable in a personal virtual coach environment | 76 | 44.4 | 0 | 30.3 | Future chatbots should include:  1. Getting insights into emotions and the process 2. A new view on “wrong eating behavior” 3. Preventing recurrence 4. Regulating emotions 5. Expelling pessimism 6. Dialectic coaching strategies |
| Fadhil, et al., 2017 [39] | To propose a chatbot system to promote healthy and sustainable eating behavior to prevent weight gain in adults | NS | NS | NS | NS | Described how AI-chatbots delivering nutrition education could overcome current limitations of similar mHealth solutions by being able to reach a broad audience on messenger apps and automate personalized messages to improve efficiency of healthcare resource allocation. |
| Gardiner, et al., 2017 [26] | To evaluates the feasibility of using an Embodied Conversational Agent (ECA) to teach lifestyle modifications to urban women. | 61 | 35 | 0 | 28 | It is feasible to use an ECA to promote health behaviors on stress management and healthy eating among urban women. ECA users significantly increased the average amount of fruit servings they ate compared to women using patient information sheets. |
| Hassoon, et al., 2020 [44] | To determine if using an AI-agent delivered by smart speaker (Amazon Echo) increases physical activity among overweight or obese cancer survivors compared to autonomous SMS or standard health education publications. | 42 | 62.1 | 1 | 32.9 | Using AI voice-assist could deliver scalable individualized behavioral health coaching to increase physical activity. After 4-week intervention, only group (A) achieved significant increase in physical activity and peaked around 10k steps/day each week. |
| Holmes, et al., 2019 [25] | To identify the needs of individuals who are maintaining weight loss in order to design and develop WeightMentor, a weight loss maintenance chatbot. | 15 | Range: 41-45 | 50 | Median: 25.3 | Individuals who are maintaining weight loss are aware of their limitations and capabilities, and will need support that is convenient and practical to track their progress and have an engaging personality. |
| Huang, et al., 2019 [34] | To present an overview of the development and implementation of “Smart Wireless Interactive Healthcare System” (SWITCHes) to facilitate objective data reception and transmission in a real-time manner. | NS | NS | NS | NS | A health chatbot-supported SWITCHes solution was developed to facilitate real-time objective data reception and transmission for healthcare professional to offer more accurate medical advice to users. |
| Kowatsch, et al., 2017 [35] | To assess enjoyment, ease of use, usefulness and the intention to use the adapted app MobileCoach and to identify and address major usability problems with the app | 11 | 12.6 | 18 | NS | The chat app was well-received on perceived ease of use (PEU), perceived enjoyment (PEN), perceived usefulness (PU) and intention to use (IU). |
| Kowatsch, et al., 2021a [29] | To propose, implement, and assess the novel coaching concept of hybrid ubiquitous coaching (HUC). | 35 | 35 | 34 | NS | Patients perceived HUC to be useful, easy to use, and enjoyable, preferred it to state-of-the-art approaches, and expressed their intentions to use it. Suggested improvements included “more specific feedback,” “more helpful instructions,” “coach should look more like a physiotherapist. Patients built a working alliance with the CA. |
| Kowatsch, et al., 2021b [29] | To propose, implement, and assess the novel coaching concept of hybrid ubiquitous coaching (HUC). | 11 | Range: 20-49 | 45 | NS | Physiotherapists indicated that patients could be better supported with the HUC and would intend to use it for treatment sessions. However, they were skeptical of whether HUC could improve the quality of the treatment and adherence though it has potential to strengthen the working alliance between physiotherapists and patients. |
| Kowatsch, et al., 2021c [29] | To propose, implement, and assess the novel coaching concept of hybrid ubiquitous coaching (HUC). | 15 | 37 | 4 | NS | AR–based CA helped participants to better understand how to do the exercise correctly. 3 patients wanted to personalize the CA (e.g, adjust the speed and the movement parameters to account for varying abilities, individually decide the appearance of the coach. 2 patients mentioned a preference for a real person and not an animated character (eg, “looking a bit more human-like, not such a computer-figure”). 2 suggested adding more detailed real-time feedback and reminders and elements of gamification (eg, rewards for regularity, real-time reminders, and accuracy). |
| Kowatsch, et al., 2021 [29] | To propose, implement, and assess the novel coaching concept of hybrid ubiquitous coaching (HUC). | 1 | NS | NS | NS | The participant felt that the AR-based CA was enjoyable and motivating and preferred the HUC to a human personal trainer, as it was perceived as being “more relaxing.” Variations of the WhatsApp-based motivational messages from the smartphone-based CA and feedback on the execution accuracy by the AR-based CA helped the patient with adherence. A more dynamic intervention program that includes longer sessions and variations in the exercises was needed. |
| L'Allemand, et al., 2018 [40] | To test a novel design of a health app for overweight adolescents, whether it supports their motivation to participate in a lifestyle intervention including relaxation and activity exercises. | 22 | 14.2 | 61 | 2.56 (BMI-SDS) | High compliance with the app over half a year may be explained by the rewarding game system, the peer character and the perceived usefulness integrated in the personalized smartphone. |
| Sandri, et al., 2019 [42] | To review the digital technology used in prevention of obesity and present the proposed STOP project that integrates state-of-the-art wearable technology, chatbot, gamification data fusion, and machine learning with the aim to provide personalized supportive feedback for preventing obesity and maintaining healthy weight. | NS | NS | NS | NS | Machine learning plays an important role in data fusion, analytics, and providing optimal messaging tailored design to support healthy weight. |
| Stasinaki, et al., 2021 [36] | To test the hypothesis that the Path-Mate2 (PM) intervention would result in a reduction in BMI-SDS after 1 year, compared to a control group (CON) receiving the usual standardized multi-component BCI. | 41 | 13.6 | 58 | 2.5 (BMI-SDS) | When the intervention focus is on weight reduction, an intensive behavior change intervention with more contact hours with healthcare professionals and longer or intermittently repeated periods of app usage may be more appropriate than using PM alone. BMI-SDS decreased in 92% of CON and 61% of the PM group between T0 and T1; from T0 to T2 a decrease in BMI-SDS was observed in 64% of CON and in 59% of the PM group, without significant group differences. |
| Stein, et al., 2017 [37] | To evaluate weight loss, changes in meal quality, and app acceptability among users of the Lark Weight Loss Health Coach AI (HCAI). | 70 | 47 | 26 | 37 | AI health coach is associated with weight loss comparable to in-person lifestyle interventions. It can also encourage behavior changes and have high user acceptability. Percentage of healthy meals increased by 31%. |
| Stephens, et al., 2019 [45] | To assess the feasibility of integrating Tess in behavioral counseling of adolescent patients coping with weight management and prediabetes symptoms. | 23 | 15.2 | 43 | NS | Results highlight the feasibility and benefit of support through AI to continue the therapeutic interaction outside office hours while maintaining patient satisfaction and aid in burnout reduction while delivering more patient support. |
| Thompson, et al., 2019 [46] | To examine the pros and cons of using a behavioral coaching social chatbot, Tess, to extend a multicomponent pediatric obesity intervention for adolescents. | NS | NS | NS | NS | Although social chatbots offer an interesting and novel method for promoting round-the-clock support, important issues and decisions must be carefully considered during the design phase to help ensure a safe environment for a vulnerable population. |
| Wu, et al., 2020 [58] | To introduce a chat-bot based approach that can meaningfully and intelligently engage with platform users, extracts information about user’s characteristics and needs, and enables a more personalized and more effective health intervention. | NS | NS | NS | NS | Gathered and analyzed data could be used to encourage healthier nutrition, and gather feedback and support on their methods for improving their eating and physical activity habits by way of the chatbot. |

**Notes:** BMI=body mass index; NS=non-specified; JITAI=just-in-time-adaptive intervention; AI=artificial intelligence
