# Supplementary material for: The Use of Artificial Intelligence–Based Conversational Agents (Chatbots) for Weight Loss: Scoping Review and Practical Recommendations
Source: JMIR Med Inform. 2022 Apr 13;10(4):e32578. doi: 10.2196/32578 (PMC9047740; doi:10.2196/32578)
Supplement: Multimedia Appendix 4 [file medinform_v10i4e32578_app4.docx]

**Appendix 4:** Use cases, names, delivery modes, deployment devices, wearables/internet-of-things and behavioral framework of the conversational agents used in the included studies (n=23).

| **Author, year** | **Use case** | **Chatbot name** | **Chatbot delivery mode** | **Chatbot deployment device** | **Wearables/IOT** | **Behavioral framework** |
| --- | --- | --- | --- | --- | --- | --- |
| Addo, et al., 2013 | Promote healthy diet and exercise | NAO humanoid robot | Speech | Humanoid robot | Kinect (motion sensor), smart refrigerator, activity tracker | Motivational interviewing |
| Asensio-Cuesta, et al., 2021a | Obesity risk self-assessment and lifestyle data collection | Wakamola chatbot (Telegram) | Text | Smartphone | NS | NS |
| Asensio-Cuesta, et al., 2021b | Obesity risk self-assessment and lifestyle data collection | Wakamola chatbot (Telegram) | Text | Smartphone | NS | NS |
| Asensio-Cuesta, et al., 2021c | Obesity risk self-assessment and lifestyle data collection | Wakamola chatbot (Telegram) | Text | Smartphone | NS | NS |
| Bardus, et al., 2018 | Promote healthy diet and exercise | WaznApp | Text | Smartphone | Activity tracker | Taxonomy of behavior changes techniques |
| Dol, et al., 2021 | Promote healthy diet | NS | NS | NS | NS | Dialectic behavior therapy |
| Fadhil, et al., 2017 | Promote healthy diet and exercise | NS | Text | NS | NS | Efficiency Model of Support |
| Gardiner, et al., 2017 | Promote healthy diet, exercise and stress management | Gabby | Text; ECA | Web browser | NS | Motivational interviewing; Mindfulness Based Stress Reduction |
| Hassoon, et al., 2020 | Promote exercise | NS | Speech | NS | NS | NS |
| Holmes, et al., 2019 | Promote healthy diet and exercise | WeightMentor (Facebook messenger) | Text | Smartphone, laptop or tablet | NS | NS |
| Huang, et al., 2019 | Promote healthy diet and exercise | SWITCHes | Speech & text | Smartphone | NS | Carver and Scheier’s control theory |
| Kowatsch, et al., 2017 | Promote exercise | MobileCoach | Text | NS | NS | NS |
| Kowatsch, et al., 2021a | Promote exercise | NS | Text and speech; AR-based embodied holographic instructor | Smartphone | AR glasses | Taxonomy of behavior changes techniques |
| Kowatsch, et al., 2021b | Promote exercise | NS | Text and speech; AR-based embodied holographic instructor | Smartphone | AR glasses |  |
| Kowatsch, et al., 2021c | Promote exercise | NS | Text and speech; AR-based embodied holographic instructor | Smartphone | AR glasses |  |
| Kowatsch, et al., 2021d | Promote exercise | NS | Text and speech; AR-based embodied holographic instructor | Smartphone | AR glasses |  |
| L'Allemand, et al., 2018 | Promote exercise and stress management | NS | Text | Smartphone | 'Sensor' | NS |
| Sandri, et al., 2019 | Promote healthy diet and exercise | NS | Text | Smartphone | NS | NS |
| Stasinaki, et al., 2021 | Promote exercise and stress management | PathMate2 | Text | Smartphone | NS | NS |
| Stein, et al., 2017 | Promote healthy diet and exercise | Lark Weight Loss Health Coach AI (HCAI) | Text | NS | ‘Wearables’ | CBT |
| Stephens, et al., 2019 | Promote healthy diet | Tess | Speech & text | NS | NS | CBT, emotionally focused therapy, motivational interviewing. |
| Thompson, et al., 2019 | Promote healthy diet | NS | Speech & text | NS | NS | NS |
| Wu, et al., 2020 | Promote healthy diet and exercise | NS | NS | NS | Activity tracker and smart watches | NS |

**Notes:** CA=conversational agent; IOT=internet of things; NS=non-specified; ECA=embodied conversational agent; AR=augmented reality; CBT=cognitive behavioral therapy
