# Supplementary material for: The Use of Artificial Intelligence–Based Conversational Agents (Chatbots) for Weight Loss: Scoping Review and Practical Recommendations
Source: JMIR Med Inform. 2022 Apr 13;10(4):e32578. doi: 10.2196/32578 (PMC9047740; doi:10.2196/32578)
Supplement: Multimedia Appendix 5 [file medinform_v10i4e32578_app5.docx]

**Appendix 5.** Architecture/descriptions and core functions of conversational agents used in the included studies (n=23).

| **Author, year** | **Architecture/Descriptions** | **Personalized recommendations** | **Motivational messages** | **Gamification** | **Sentiment analysis** |
| --- | --- | --- | --- | --- | --- |
| Addo, et al., 2013 | 1. Collective intelligence - data mining of physical activity and healthy diet choices 2. Discovering Profile Segments - provide adaptive motivational messages and suggestions suitable for each profile segment 3. Motivational Interviewing - stimulate readiness to change  4. Human Robot Interaction - Humanoid (physical) and Kinect (virtual) applications implement emotion detection features to interpret and simulate human verbal and non-verbal cues (e.g. facial expressions, eye contact, gaze direction, speech and tone recognition and hand gestures) 5. Virtual environment - addictive interactive games that encourage physical exercise and communicating with others 6. Spoken Language Understanding Service 7. Natural Language Sentiment Analysis | Y | Y | Y | Y |
| Asensio-Cuesta, et al., 2021a | 1. Gamification (score points for goals achieved) 2. Socialization 3. Feedback to user (obesity risk with graphical representation)  4. Emotional engagement: personification of chatbot through Wakamola character | Y | Y | Y | NS |
| Asensio-Cuesta, et al., 2021b |  | Y | Y | Y | NS |
| Asensio-Cuesta, et al., 2021c |  | Y | Y | Y | NS |
| Bardus, et al., 2018 | Collected data are used to generate interactive conversations every time individuals use the app. The app sends notifications to prompt the users to review their activity throughout the day. Users may receive information about health consequences, help with developing problem-solving skills (e.g. providing alternative healthier food options), emotional support and positive reinforcement. | Y | Y | N | NS |
| Dol, et al., 2021 | NS | Y | Y | N | NS |
| Fadhil, et al., 2017 | Data including emotional and choice dynamics are collected to determine user intent and perform sentiment analysis, to provide a more tailored coaching towards a sustainable healthy diet. The bot can be trained to understand the user state regarding a given plan, trigger the right action and notify a healthcare expert (e.g., nutritionist) when needed.  The **Intent Detection** first categorizes a request into predefined intents and prescribes an action that defines the desired outcome. The **Role Detection** then assigns predefined categories to entities of particular type. The **Entity Resolution** matches the identified entity with a real-world object or concept and **Question Answering** identifies the best answer for the request based on knowledge base or acquired data. Finally, the **Dialogue Management** tracks the context of the conversation and formulates the appropriate response to the user. | Y | Y | NS | Y |
| Gardiner, et al., 2017 | The system is comprised of a networked server with a conversational database responding to patient’s interactions and a patient education system. The scripts included motivational interviewing dialogue, dialogue to simulate shared decision-making interactions, and additional longitudinal interaction using techniques such as goal setting, problem-solving, tips, and homework. | Y | Y | NS | NS |
| Hassoon, et al., 2020 | NS | Y | NS | NS | NS |
| Holmes, et al., 2019 | Messages sent through messenger trigger an event in a Webhook, and messages are then passed to a Facebook-Messenger enabled app. Messages received by the backend will either be handled directly within the Node.js code or passed to a **Dialogflow agent.** 4 types of conversations: ordinary (casual and unconstrained), service (constrained by rules and roles), teaching (between teacher/instructor and student), counselling (led by counselee, who seeks advice from counsellor). | Y | Y | NS | NS |
| Huang, et al., 2018 | Contains two main components: an interactive web-based dashboard and a mobile app. Upon start-up, individuals set goals which SWITCHes app will use to create a feasible plan for self-monitoring. Users can talk to the health chatbot and get real-time information, or take a bot’s personalized recommendations (e.g. diet and exercise tracking and advice, eating tips, proactive weight prediction). | Y | NS | NS | NS |
| Kowatsch, et al., 2017 | Participants first interacted with the chatbot through various chat-based photo, physical activity and quiz interactions. A generic dashboard view summarizes key statistics of the envisioned behavioral health interventions for self-monitoring purposes (e.g. steps achieved per day, intervention progress or goals achieved). | NS | NS | NS | NS |
| Kowatsch, et al., 2021a | Smartphone-based CA reminds the patient to do the home exercises, motivates the patient via the smartphone, and emphasizes the benefits of performing the exercise by providing psychoeducational material. AR-based CA delivers real-time exercise by demonstrating and guiding the patient through the exercise, monitoring the progress and giving real-time feedback. It also informs and emphasizes essential aspects of how to perform the exercise correctly. The AR-based CA counts the number of sets and repetitions out loud and provides both visual and auditory feedback on the exercise execution after a completed set of exercises. Feedback was based on comparing patient data from the AR system with data about the physiotherapist performing the same exercise. | Y | Y | NS | NS |
| Kowatsch, et al., 2021b |  | Y | Y | NS | NS |
| Kowatsch, et al., 2021c |  | Y | Y | NS | NS |
| Kowatsch, et al., 2021d |  | Y | Y | NS | NS |
| L'Allemand, et al., 2018 | An open source platform with a text-based healthcare chatbot (THCB) within a mobile chat app with game character designed for Android smartphones. | NS | NS | Y | NS |
| Sandri, et al., 2019 | The proposed intelligent chatbot integrates data collected and interpret the user’s emotion to provide tailored feedback. It prevents situations where uncertainty takes control of the dialog flow; tedious, monotonous, and boring conversations and conversations that tries to substitute health professionals or doctors. It also tries to include reinforcement learning (to accept user’s corrections over time to improve suitability of responses), natural language processing (to read human text and understand sentences), entity recognition (to understand that the analysed text is talking about an informative abstract category), machine learning (to learn how to respond to a user by analysing human agent responses), intent recognition (to “guess” what the user is requesting even if phrased unexpectedly) and dialog management (to follow conversation history) | Y | Y | NS | Y |
| Stasinaki, et al., 2021 | Developed with the MobileCoach open-source software for health interventions. The conversational agent chatted daily with the participants, encouraging them to achieve challenges like a number of steps per day, performing relaxing breathing exercises, taking photos of their meals, or answering quality of life questions in order to earn virtual rewards. Most of the time and to allow efficient and reproducible conversational turns, patients were able to respond to Anna/Lukas with predefined answer options. In the app dashboard, the patients could see their progress during the game. Furthermore, a dashboard overview alerted HCP, in case of lack of chat interaction during more than 2 days. | NS | NS | Y | NS |
| Stein, et al., 2017 | Conversations with patients are triggered by real-time data (e.g. dietary consumption) gathered automatically from sensors on phones, wearables, or by self-reports. Lark’s AI health coaches mimic health professionals’ empathetic health counseling through casual conversations using empathetic text-based communication and other interactive elements. It learns about users and provides personalized content. | Y | Y | NS | Y |
| Stephens, et al., 2019 | Provides personal and on-demand conversations, replies with scripted statements that have been crafted and reviewed by mental health professionals to mimic empathy and compassion. Tess delivers interventions and responds based on the individual’s reported emotion or concern. Tess also asks for feedback in order to deliver more personalized support (e.g., adjusting interventions provided based on level of self-reported usefulness). Tess learns from every interaction on an individual and group level. After each intervention, Tess asks the user if the suggestions provided were helpful. If yes, then Tess will deliver interventions that align with that topic or modality. If no, Tess will try something different to give the user alternative intervention options. | Y | Y | NS | Y |
| Thompson, et al., 2019 | NS | Y | Y | NS | NS |
| Wu, et al., 2020 | Collected chat data will be used for natural language processing and behavior analysis, along with other available data, to create a customized user model to encourage healthier nutrition. Uses Microservice architecture (by doing the broken-down process, the overall structure becomes more loosely coupled which gain more readability and maintainability) and containerization (allows developers to wrap the application along with the specified configuration files, libraries and required dependencies together into a container which can run in any computing environment). | Y | Y | NS | Y |

Notes: NS=non-specified
