# Supplementary material for: The Use of Artificial Intelligence–Based Conversational Agents (Chatbots) for Weight Loss: Scoping Review and Practical Recommendations
Source: JMIR Med Inform. 2022 Apr 13;10(4):e32578. doi: 10.2196/32578 (PMC9047740; doi:10.2196/32578)
Supplement: Multimedia Appendix 6 [file medinform_v10i4e32578_app6.docx]

**Appendix 6:** Parameters collected from users to develop conversational agent-based weight loss interventions (n=23).

| **Author, year** | **Socio-**  **demographics** | **Anthropometrics** | **Diet** | **Physical activity** | **Mental health** | **Sleep** | **Clinical profiles** |
| --- | --- | --- | --- | --- | --- | --- | --- |
| Addo, et al., 2013 | NS | NS | Smart Refrigerator Appliance that tracks diet choices | Data collected from wearable sensors and smartphones; | NS | NS | NS |
| Asensio-Cuesta, et al., 2021a | Gender, age, level of education, marital status, number of at home | Weight, height | Short questionnaire on frequency of dietary intake: frequency of eating 50 food types | IPAQ-SF | NS | sleep hours | Existence of a previous diagnosis of hypertension, diabetes, high cholesterol, or cardiovascular disease, smoking status |
| Asensio-Cuesta, et al., 2021b | Gender, age, level of education, marital status, how many people are at home | Weight, height | Short questionnaire on frequency of dietary intake: frequency of eating 50 food types | IPAQ-SF | NS | sleep hours | Existence of a previous diagnosis of hypertension, diabetes, high cholesterol, or cardiovascular disease, smoking status |
| Asensio-Cuesta, et al., 2021c | Gender, age, level of education, marital status, how many people are at home | Weight, height | Short questionnaire on frequency of dietary intake: frequency of eating 50 food types | IPAQ-SF | NS | sleep hours | Existence of a previous diagnosis of hypertension, diabetes, high cholesterol, or cardiovascular disease, smoking status |
| Bardus, et al., 2018 |  | Weight, height | User-inputted food meals | User-inputted physical activities, data collected from wearable sensors and smartphones; | NS | Phone motion sensors and/or Apple Watch detected | NS |
| Dol, et al., 2021 | NS | NS | NS | NS | NS | NS | NS |
| Fadhil, et al., 2017 | NS | NS | NS | NS | NS | NS | NS |
| Gardiner, et al., 2017 | Age, race, ethnicity, education, work status, income | Weight, height | Adapted questions from the National Health and Nutrition Examination Survey (NHANES) | Short Form Health Survey (SF-12); Stanford Patient Education Research Center’s Exercise Behaviors Questionnaire; Self-Efficacy for Exercise Scale (SEE) | Patient Health Questionnaire (PHQ-9); Perceived Stress Scale (PSS) | NS | Health conditions, medication use, smoking status, alcohol use, and pain levels |
| Hassoon, et al., 2020 | NS | NS | NS | NS | NS | NS | NS |
| Holmes, et al., 2019 | NS | NS | NS | NS | NS | NS | NS |
| Huang, et al., 2019 | NS | Not detailed | Not detailed | Not detailed | NS | NS | NS |
| Kowatsch, et al., 2017 | NS | NS | NS | NS | NS | NS | NS |
| Kowatsch, et al., 2021a | NS | NS | NS | NS | NS | NS | NS |
| Kowatsch, et al., 2021b | NS | NS | NS | NS | NS | NS | NS |
| Kowatsch, et al., 2021c | NS | NS | NS | NS | NS | NS | NS |
| Kowatsch, et al., 2021d | NS | NS | NS | NS | NS | NS | NS |
| L'Allemand, et al., 2018 | NS | NS | NS | NS | NS | NS | NS |
| Sandri, et al., 2019 | NS | Weight, height | Nutrition information provided retailers and self-reports; USDA Food Composition Database (https://ndb.nal.usda.gov/ndb/) and MyFood (https://myfood.okkam.it/) datasets | Self-reports, data collected from wearable sensors and smartphones; | NS | NS | Heart rate, blood pressure |
| Stasinaki, et al., 2021 | NS | Weight, height, body composition (muscle and fat), waist-to-height ratio | NS | Dordel- Koch-Test: strength, agility, flexibility, endurance, balance | Stress parameters (Plasma cortisol, skin conductance response) | NS | Heart rate, blood pressure |
| Stein, et al., 2017 | Age, gender | Weight, height | NS | Self-reports; data collected from wearable sensors and smartphones. | Self-reports and automatic detection by the user’s mobile phone. | age, gender | NS |
| Stephens, et al., 2019 | NS | NS | NS | NS | NS | NS | NS |
| Thompson, et al., 2019 | NS | NS | NS | NS | NS | NS | NS |
| Wu, et al., 2020 | NS | Weight, height | Nutrition information provided retailors and self-reporting (collected by grocery calories identification via a mobile app), meal calories estimation and self- reports | Self-reporting; data collected from wearable sensors and smartphones; | NS | NS | Heart rate, blood pressures |

Notes: NS=non-specified; IPAQ-SF= International Physical Activity Questionnaire-Short Form
