# Supplementary material for: The Use of Artificial Intelligence–Based Conversational Agents (Chatbots) for Weight Loss: Scoping Review and Practical Recommendations
Source: JMIR Med Inform. 2022 Apr 13;10(4):e32578. doi: 10.2196/32578 (PMC9047740; doi:10.2196/32578)
Supplement: Multimedia Appendix 7 [file medinform_v10i4e32578_app7.docx]

**Appendix 7:** Engagement, Satisfaction and human-CA emotional connection described in the included studies (n=23).

| **Author, year** | **Engagement** | **Satisfaction** | **Techniques improve trust, build rapport and emotional connection (besides giving positive reinforcements)** | **Improvements suggested** |
| --- | --- | --- | --- | --- |
| Addo, et al., 2013 | NS | 60% willing to welcome the Human-Robot Interaction in real-life; 80% willing to interact with the robot in a lab-setting periodically while welcoming wearable and mobile physical activity tracking devices as part of their everyday routine. | Humanoid (physical) and Kinect (virtual) applications implements emotion detection features to interpret and simulates human verbal and non-verbal cues. Robot exhibited believable behaviors including emotion display and some form of social cues during the interaction. | Concerns with privacy. |
| Asensio-Cuesta, et al., 2021a | Time of connection per user ranged from 26.31 to 36.27 minutes; number of connections per user ranged from 1.0 to 1.07 indicating a low user engagement after the first use. | NS | Uses **colloquial tone** to recall human-to-human interaction and improve user’s emotional connection. Includes **emoji** in the messages that contribute to a more realistic and friendly conversation | NS |
| Asensio-Cuesta, et al., 2021b | NS | System usability Scale (SUS) questionnaire (n=61): Only 46% of participant´s SUS score was 51 or higher, corresponding to an “ok” level of perceived usability | Positively-framed words e.g. the word “risk” changed to “status” for overweight assessment. **Personification** of chatbot through Wakamola character; introduction of **human-like cues** (eyes, mouth, expressions of effort and happiness) and use of **emoji t**o create a more realistic and friendly conversation. | Dependency on the Telegram platform, diet section contains too many questions. Number of chatbot messages needs to be limited to avoid user fatigue and abandonment.  Needed advice on diet and physical activity (general and personalized), tracking (diet, physical activity, calories consumed), community, sharing progress, positive messages, information about nutrition and healthy habits, success stories, syncing with activity bracelets, and rewards for improving, among others. Should include information about the app objectives and comes from a reliable source. |
| Asensio-Cuesta, et al., 2021c | NS | NS | Positively-framed messages e.g. the word “risk” changed to “status” for overweight assessment. **Personification** of chatbot through Wakamola character; introduction of **human-like cues** (eyes, mouth, expressions of effort and happiness) and **emoji t**o create a more realistic and friendly conversation helps users to empathize with it. | NS |
| Bardus, et al., 2018 | NS | NS | Positive enforcement, motivational messages, citing "credible source" | NS |
| Dol, et al., 2021 | NS | NS | Dialectic coaching strategy:  **Validation** (especially compliment) of emotions and behaviour: Empathy, empowerment, non-judgemental **Focus on change:** Help to sense and understand emotions: gain more knowledge about their own emotions and feelings and how to deal with them | Needed practical tips on how to act differently next time. CA should provide encouragement to act and ask more to gain better insight into an individual’s eating behaviour, helping them reconstruct what just happened and make a relapse prevention plan. |
| Fadhil, et al., 2017 | NS | NS | NS |  |
| Gardiner, et al., 2017 | Median time of engagement: 52 mins (interquartile range [IQR] = 101.4, N = 31); number of connections: 5 times; average time spent per individual session: 12.1 mins | 69% utilized suggestions from Gabby to improve healthy eating, 52% to increase physical activity, and 70% to manage stress | African American female character programmed to deliver **culturally apt** patient strategies and health information (e.g. recommending specific popular recipes or foods, mentioning prayer or spiritual traditions). Language used was dynamically composed, based on each patient’s data (e.g. name, gender, history of past interactions and responses). Empowering, relatable, fast, reliable, and credible source of information. | Challenges with the sound and quality of Cas voice (too robotic and slow), time commitment to use the system, and program accessibility (devices access). “No accountability, no checking to see if you made improvements” and “Sheets were easy to ignore”. |
| Hassoon, et al., 2020 | NS | NS | NS | NS |
| Holmes, et al., 2019 | NS | NS | WeightMentor prototype was engaging, friendly and sometimes funny, without being too pushy. | Too detailed or cumbersome, reduced convenience. Apps should be convenient and support progress tracking. Personal messages should be specific and relevant. |
| Huang, et al., 2019 | NS | NS | NS | NS |
| Kowatsch, et al., 2017 | NS | Successfully completed ~ 61% of the daily intervention tasks. Perceived enjoyment, usefulness: and ease of use: median=6-7 | NS | NS |
| Kowatsch, et al., 2021a | NS | NS | Empathetic and human-like AR-based CA | NS |
| Kowatsch, et al., 2021b | NS | NS | AR-based CA was scaled up to be human-sized and adapted in appearance to look like a physiotherapist to provide a professional and natural human-like style shown to increase intention to use. AR-based CA increases the attachment bond and dimension of a working alliance by literally giving high fives or showing the patient a funny dance move when a set or training session is completed. The AR-based CA was perceived as an additional motivator due to its personal, interactive, and playful approach. The exercise guidance by the life-sized CA, including the real-time feedback (eg, automatic counting of exercise repetitions), was also perceived as an improvement to the status quo. | NS |
| Kowatsch, et al., 2021c | NS | NS | NS | NS |
| Kowatsch, et al., 2021d | NS | Adherence rate of 92% (11/12 sessions; 330/360 repetitions; 33/36 sets) and a substantial increase in exercise accuracy during the 4 weeks | NS | NS |
| L'Allemand, et al., 2018 | After 4 months, ~70% of the patients had >4 conversational turns per day, 37% of the daily challenges were completed successfully. | NS | Peer character | NS |
| Sandri, et al., 2019 | NS | NS | NS | NS |
| Stasinaki, et al., 2021 | After half a year, 51% of the PM participants were still using the App and average daily app usage rate: 71.5% | 54% were active on achieving challenges, average adherence rate was 57.2% during the first 5.5 months of the study. | NS | NS |
| Stein, et al., 2017 | Average duration of app use: 15 (SD 1.0) weeks, and users averaged 103 sessions each. | Satisfaction score of 87 out of 100, net promoter score = 47 | Expresses empathy through a “subsequent desire to help” through conversations around the event or feeling. The conversations deliver messages that provide both strategic help and emotional support. | NS |
| Stephens, et al., 2019 | The longest conversation between Tess and a teen patient was 1 hr and 13 min long,while the shortest conversations fell between 4 and 7 s; Average duration of conversations: ~12.5 min | Adolescent patients reported experiencing positive progress toward their goals 81% of the time and usefulness ratings 96% of the time. | NS | NS |
| Thompson, et al., 2019 | NS | NS | NS | Unclear what safeguards are in place to prevent misinterpretation of information provided by the user or to protect against Tess going off-script and providing incorrect or potentially harmful information. Future development and refinement of Tess will likely require annotation of interactive sequences by professionals and a careful examination of procedures and response algorithms to ensure safeguards are in place to help ensure content fidelity and accuracy. |
| Wu, et al., 2020 | NS | NS | Feedback provided are friendly, easy to understand, reliable and respect the behavioural changes on the user. | NS |

Notes: NS=non=specified, CA=conversational agent
